# Microbial Metabolite Sodium Butyrate Attenuates Cartilage Degradation by Restoring Impaired Autophagy and Autophagic Flux in Osteoarthritis Development

**DOI:** 10.3389/fphar.2021.659597

**Published:** 2021-04-09

**Authors:** Haikang Zhou, Guoqing Li, Yang Wang, Rendong Jiang, Yicheng Li, Huhu Wang, Fei Wang, Hairong Ma, Li Cao

**Affiliations:** ^1^ Department of Orthopaedics, First Affiliated Hospital of Xinjiang Medical University, Urumqi, China; ^2^ Xinjiang Uygur Autonomous Region Clinical Research Center for Orthopedic Diseases, First Affiliated Hospital of Xinjiang Medical University, Urumqi, China

**Keywords:** sodium butyrate, osteoarthritis, autophagy, apoptosis, oxidative stress

## Abstract

Osteoarthritis (OA) is a degenerative joint disease with multiple etiologies that affects individuals worldwide. No effective interventions are currently available to reverse the pathological process of OA. Sodium butyrate (NaB), a component of short-chain fatty acids (SCFAs), has multiple biological activities, including the attenuation of inflammation and anti-tumor activities in various diseases. However, whether the protective effects of NaB in OA are associated with the promotion of autophagy had not been investigated. Here, we explored the chondroprotective properties of NaB in an interleukin (IL)-1β-induced inflammatory chondrocyte model and an anterior cruciate ligament transection (ACLT) mouse model. Hematoxylin and eosin (HE), Safranin O, and immunohistochemical staining were performed to evaluate the effects of NaB treatment on articular cartilage. An optimal NaB dose for chondrocyte treatment was determined *via* cell counting kit-8 assays. Immunofluorescence and transmission electron microscopy were used to detect autophagy in chondrocytes. Flow cytometry was utilized to detect reactive oxygen species (ROS), cell cycle activity, and apoptosis in chondrocytes. Western blot and immunostaining were performed to evaluate the protein expression levels of relevant indicators. We found that the administration of NaB by oral gavage could attenuate cartilage degradation. In parallel, NaB treatment could enhance the activation of autophagy, increase autophagic flux, decrease extracellular matrix degradation, and reduce apoptosis by restraining inflammation, ROS production, and cell cycle arrest in IL-1β-treated chondrocytes. The protective effects of NaB could be partially abolished by the autophagy inhibitor 3-methyladenine (3-MA), which indicated that the protective effects of NaB against OA were partially governed by the enhancement of autophagy to restrain the formation of inflammatory mediators and ROS and regulate cell cycle progression and apoptosis in chondrocytes. In conclusion, NaB could attenuate OA progression by restoring impaired autophagy and autophagic flux *via* the phosphoinositide 3-kinase (PI3K)/protein kinase B (Akt)/mammalian target of rapamycin (mTOR) pathway, both *in vitro* and *in vivo*, implying that NaB could represent a novel therapeutic approach for OA.

## Introduction

Osteoarthritis (OA) is one of the most prevalent musculoskeletal diseases among older individuals, causing serious social and economic burdens (Jones et al., 2019). The incidence of OA has increased in association with the increased prevalence of obesity and the overall aging of the population. More than 28% of people over the age of 60 have been estimated to be affected by OA (Hunter and Bierma-Zeinstra, 2019), and the average cost per year associated with OA management has been estimated to account for 25–50% of a country’s GDP (Puig-Junoy and Ruiz, 2015). Although several etiologies have been verified to contribute to the risk of OA development, such as aging, obesity, and dysbiosis of the gut microbiota (GMB), no pharmacological therapy has yet been developed to alleviate the pathological processes underlying OA. The relief of symptoms using current conservative treatment options remains very limited, and total joint arthroplasty is currently the last option for the management of end-stage OA (Carr et al., 2012). Thus, the exploration of new medical therapies to alleviate, halt, or reverse OA development represents an urgent need.

The pathological processes of OA primarily revolve around the apoptosis, oxidative damage, senescence, and autophagy of chondrocytes. Autophagy, as an intracellular degradation system, maintains energy homeostasis and nutrient regulation under conditions of cellular stress (Klionsky et al., 2016), which has been validated by the adjustment of cellular homeostasis in healthy chondrocytes (Almonte-Becerril et al., 2010). Emerging evidence has indicated that autophagy shows a declining tendency after reaching a peak during the initial pathological stages of OA, and this decline in autophagy has been associated with an increase in chondrocyte apoptosis and OA aggravation (Cheng et al., 2017). Mammalian target of rapamycin (mTOR) serves a vital role in autophagy regulation (Mizushima et al., 2008). The hyperphosphorylation of mTOR is observed in human OA cartilage samples, associated with reduced autophagy and increased chondrocyte apoptosis (Zhang Y. et al., 2015). Furthermore, the cartilage-specific mTOR knockout not only enhanced autophagy and reduced cartilage degradation but also significantly reduced the apoptosis rate and synovial fibrosis development in articular cartilage (Zhang Y. et al., 2015). Therefore, the selective targeting of mTOR-mediated autophagy may represent a novel and attractive treatment for OA.

Sodium butyrate (NaB) is a short-chain fatty acid (SCFA) and a primary metabolite produced by the bacterial fermentation of undigested dietary fibers in the large intestine (Tan et al., 2014). The serum concentration of NaB has been shown to be dramatically elevated by the addition of the probiotic *Lactobacillus rhamnosus* GG or high levels of dietary fiber to the diets of mice (Lucas et al., 2018; Tyagi et al., 2018). In recent years, GMB and associated metabolites have been found to be closely associated with various multi-system diseases. Moreover, increasing evidence has suggested that the biological functions of SCFAs are closely associated with the homeostasis of bone metabolism (Zaiss et al., 2019; Li et al., 2020). Interestingly, one study revealed that the anti-tumor properties of NaB were partly mediated by the activation of autophagy (Zhang et al., 2016), whereas another study reported that the restriction of mTOR complex 1 (mTORC1) by SCFAs could enhance the activation of autophagy (Noureldein and Eid, 2018). Furthermore, recent studies demonstrated that NaB could protect chondrocytes from interleukin (IL)-1β-induced inflammation and prevent the extracellular matrix (ECM) degradation of chondrocytes *via* the regulation of the nuclear factor-κB (NF-κB) pathway (Bo et al., 2018; Pirozzi et al., 2018), However, whether the protective effects of NaB on OA is partially mediated by the promotion of autophagy has not been well-investigated, and the exact mechanisms through which NaB enhances autophagy and its potential cartilage-protective effects in OA remain unclear.

In this study, we utilized bioinformatics methods to predict possible target genes and pathways associated with NaB and which may be involved in the therapeutic mechanisms associated with NaB. The identified phosphoinositide 3-kinase (PI3K)/protein kinase B (Akt)/mTOR pathway may represent a crucial signaling pathway through which NaB protects chondrocytes and alleviates OA. We then investigated whether NaB could attenuate OA progression, including the amelioration of IL-1β-induced inflammation, reactive oxygen species (ROS) production, cell cycle arrest, and apoptosis, by restoring impaired autophagy *via* the PI3K/Akt/mTOR pathway in both an *in vitro* chondrocyte model and an *in vivo* mouse model of articular cartilage degeneration induced by anterior cruciate ligament transection (ACLT). Altogether, our results implied that NaB could represent a novel therapeutic approach for OA.

## Materials and Methods

### Butyrate-Targeted Genes Search

The interrelationship between butyrate and its correlated genes were explored and predicted by STITCH (http://stitch.embl.de/). Firstly, butyrate was put into the STITCH to select the *Homo sapiens*. After that the networks were calculated using the following settings: medium confidence score 0.4; maximum number of interactions: first shell: 0 and up to 20; second shell: 0 and up to 30. Finally, the network was exported as a tabular text output and the network of butyrate-targeted genes were reconstructed and visualized by performing the Cytoscape software.

### Osteoarthritis Related Genes Collection

Firstly, the human OA related gene set was retrieved by miRWalk 2.0. Then, we collected genes related to Osteoarthritis from MalaCards (https://www.malacards.org/card/osteoarthritis). Genes from these two databases were combined as OA related genes.

### Gene Ontology Enrichment Analysis

To determine functional categories of butyrate-targeted genes and OA related genes, Gene Ontology (GO; http://geneontology.org/) analysis was performed using the KOBAS 2.0 server. The most enriched 10 terms were selected and plotted. Biological process terms with corrected *p* value ≤0.01 were used for other analysis. Terms utilized by butyrate-targeted genes that were also associated with OA were selected.

### Reagents and Antibodies

NaB (purity ≥98%) was purchased from Aladdin® (Shanghai, China). 3-methyladenine (3-MA), and type II collagenase came from Sigma-Aldrich (MO, United States). The indicated dilutions of the following primary antibodies were used in this study for western blot (WB), immunofluorescence (IF) and immunohistochemistry (IHC) analysis: Aggrecan (13880-1-AP, Proteintech, 1:1000 for WB, 1:200 for IHC), Collagen II (28459-1-AP, Proteintech, 1:1500 for WB), MMP-3 (17873-1-AP, Proteintech, 1:1000 for WB) were purchased from Wuhan Proteintech Group (Wuhan, China). Antibodies against Bax (#5023, CST, 1:1000 for WB, 1:100 for IHC), Bcl-2 (#3498, CST, 1:1000 for WB), LC3 (#43566, CST, 1:1000 for WB, 1:100 for IF, 1:200 for IHC), Beclin-1 (#3495, CST, 1:1000 for WB), SQSTM1/p62 (#23214, CST, 1:1000 for WB, 1:50 for IF, 1:100 for IHC) were acquired from Cell Signaling Technology (Danvers, MA, United States). PI3K (ab140307, Abcam, 1:2000 for WB), p-PI3K (ab154598, Abcam, 1:1000 for WB, 1:100 for IHC), Akt (ab8805, Abcam, 1:2500 for WB), p-Akt (ab38449, Abcam, 1:1000 for WB, 1:100 for IHC), mTOR (ab134903, Abcam, 1:5000 for WB), p-mTOR (ab109268, Abcam, 1:1000 for WB, 1:200 for IHC), COX-2 (ab179800, Abcam, 1:1000 for WB) and GAPDH (ab8245, Abcam, 1:7000 for WB) were from Abcam (Cambridge, MA, United States).

### Primary Chondrocytes Isolation and Culture

The 2 week-old C57BL/6 mice were euthanized and primary chondrocytes were isolated from the mice knee cartilages according to the previous literature (Zheng et al., 2018b). Then, we cut the cartilages into pieces and digested with 2 mg/ml of collagenase II in DMEM/F12 (Gibco, Grand Island, NY, United States) medium at 37°C on shaking table for 4 h. Finally, the digested single cell suspension was centrifuged at 1,000 rpm for 5 min, then the supernatant was discarded and washed with PBS for three times. After that primary chondrocytes were resuspended and inoculated in a humidified incubator with 5% CO_2_ at 37°C. Cells from the second passage were used for all experimental studies.

### Cell Viability Assay

CCK-8 assays (Dojindo Co., Kumamoto, Japan) were used to evaluated the cytotoxicity of NaB on chondrocytes. In berif, chondrocytes were seeded into 96-well plates (1 × 10^4^ cells/well) with 100 μl DMEM/F12 medium per well in the presence of NaB at gradient concentrations (31.25, 62.5, 125, 250, 500, and 750 μM) for 24 h. In the other group, chondrocytes were pretreated with NaB (31.25, 62.5, 125, 250 μM) for 2 h, then co-incubated with IL-1β (10 ng/ml) for a further 24 h. At the end of the intervention time, 10 μl CCK-8 solution was added into each well, incubated at 37°C for 2 h. Finally, absorbance of each well was measured at 450 nm by a Microplate reader (Thermo, United States).

### Western Blot Analysis

Cytosolic proteins were extracted from the chondrocytes of different treatment groups by RIPA lysis buffer containing protease inhibitors. Equal amount of protein (20 μg) was separated on SDS-PAGE gels and transferred onto a nitrocellulose membrane. The target proteins were detected by primary antibodies following with corresponding horseradish peroxidase-conjugated secondary antibodies. Finally, the chemiluminescent signals of protein bands were visualized using a Gel Doc 2000 Imager (Bio-Rad, United States).

### Immunofluorescence

Chondrocytes from different treatment groups were washed once in phosphate-buffered saline (PBS) followed by fixation for 15 min in 4% paraformaldehyde and permeabilization for 15 min in 0.4% Triton X-100. Chondrocytes were blocked for 30 min with 5% goat serum, washed three times in PBS, and incubated overnight at 4°C with antibodies against microtubule-associated protein 1A/1B-light chain 3 (LC3)-II and sequestosome 1 (SQSTM1)/p62. After washing three times with PBS, the cells were incubated with Alexa Fluor488-or Alexa Fluor594-conjugated secondary antibodies for 1.5 h at 37°C, followed by 4′,6-diamidino-2-phenylindole (DAPI) incubation for 3 min to stain the nuclei. Finally, a fluorescent microscope (Olympus Inc., Tokyo, Japan) was used to visualize the samples, and fluorescence intensity was evaluated by ImageJ software, version 2.1 (Bethesda, MD, United States).

### Flow Cytometric Analysis of Cell Apoptosis

A flow cytometric apoptosis kit was used to detect the apoptosis rate of chondrocytes across multiple intervention groups. The collected chondrocytes were resuspended in Annexin V-binding buffer and then stained with 5 µl phycoerythrin (PE)-labeled Annexin V and 5 µl **7**-aminoactinomycin D (7-ADD) for 15 min at 37°C in the dark. The stained cells were assessed with a FACSAria^™^ II flow cytometer (BD Biosciences) within 1 h of staining.

### Intracellular Reactive Oxygen Species Measurement

An ROS assay kit (Solarbio Science, Beijing, China) was applied to evaluate the intracellular ROS level of chondrocytes in different intervention groups, followed by flow cytometry. The collected chondrocytes were incubated with 10 μmol/L 2′,7′-dichlorofluorescein diacetate (DCFH-DA) at 37°C for 20 min and then washed twice with serum-free medium. The ROS levels of different groups were detected by flow cytometry (BD Biosciences, United States).

### Cell Cycle Detection

The chondrocyte cell cycle was evaluated using a cell cycle kit (Solarbio, China). Chondrocytes from different intervention groups were washed once with PBS and centrifuged at 1,500 rpm for 5 min to collect the cells. The cell density was adjusted to 1 × 10^6^ cells/ml. A 1 ml aliquot of the cell suspension was centrifuged, and the supernatant was discarded, followed by the addition of pre-cooled 70% ethanol and incubation at 4°C overnight for fixation. After washing three times with PBS, 100 µl RNase A solution was added to the cell pellet to resuspend the cells and incubated in a 37°C water bath for 30 min, followed by the addition of 400 µl propidium iodide (PI) staining solution, which was mixed and incubated at 4°C for 30 min in the dark. Finally, the chondrocyte samples were analyzed by flow cytometry (BD, United States).

### Transmission Electron Microscopy

Chondrocytes from different treatment groups were fixed in 2.5% glutaraldehyde overnight at 4°C. Subsequently, 1% osmium tetroxide was used for post-fixation at 4°C for 2 h. The chondrocytes were then dehydrated through an acetone gradient and embedded into Araldite. The samples were sectioned and observed by transmission electron microscopy (TEM, Hitachi, Tokyo, Japan).

### Animals

All experiments and therapies were conducted in accordance with approval from the Institutional Animal Care and Use Committee of the First Affiliated Hospital of Xinjiang Medical University (protocol number IACUC20180223-176). No clinical trial was performed for the current experiments. Three-month-old male C57BL/6J mice were purchased from Vital River (Beijing, China) and allowed to adapt for 1 week to feeding and housing conditions. Detailed surgical procedures describing the transection of the anterior cruciate ligament to establish an OA model can be found in our previous study (Mu et al., 2018). First, we performed a preliminary experiment to identify the optimal dose. Mice were randomly distributed into the sham, vehicle, and multiple NaB groups (50, 100, 150, and 200 mg/kg; *n* = 10 per group). The right knee was subjected to ACLT to establish the OA model. Subsequently, NaB or an equivalent volume of sterile saline was administered daily by oral gavage for 60 days starting on the second day after ACLT. During the formal experiment, we randomly divided mice into a sham group, a vehicle group, and an optimal NaB treatment group (*n* = 20 per group). Ten mice from each group were sacrificed at each of 30 and 60 days postoperation.

### Histological Analysis and Immunostaining

For detailed pathological staining and immunohistochemical procedures, please refer to our previous study (Mu et al., 2018). Briefly, 4-mm-thick sections of samples were prepared for hematoxylin and eosin (HE) and Safranin O staining and immunostaining. The thickness of the hyaline cartilage (HC) and the calcified cartilage (CC) were measured using HE staining. A standard protocol was used to perform immunohistochemical staining. Finally, a horseradish peroxidase streptavidin detection system (ZSGB BIO) was used to detect immunoactivity, followed by counterstaining with hematoxylin (ZSGB BIO). The histologic scoring of OA in the medial tibial plateau was calculated as described by Glasson et al. (2010).

### Statistical Analysis

The results were presented as mean ± S.D. Statistical analyses were performed using SPSS statistical software program 20.0 (IBM, Armonk, NY, United States). Data were analyzed by one-way analysis of variance (ANOVA) followed by Tukey’s test for comparison between control and treatment groups. Nonparametric data (OARSI scores) were analyzed by the Kruskal Wallis H test. Statistical significance was set at *p* < 0.05.

## Results

### Gene Ontology Terms Enriched in Both Butyrate-Targeted Genes and Osteoarthritis-Related Genes

**FIGURE 1:**
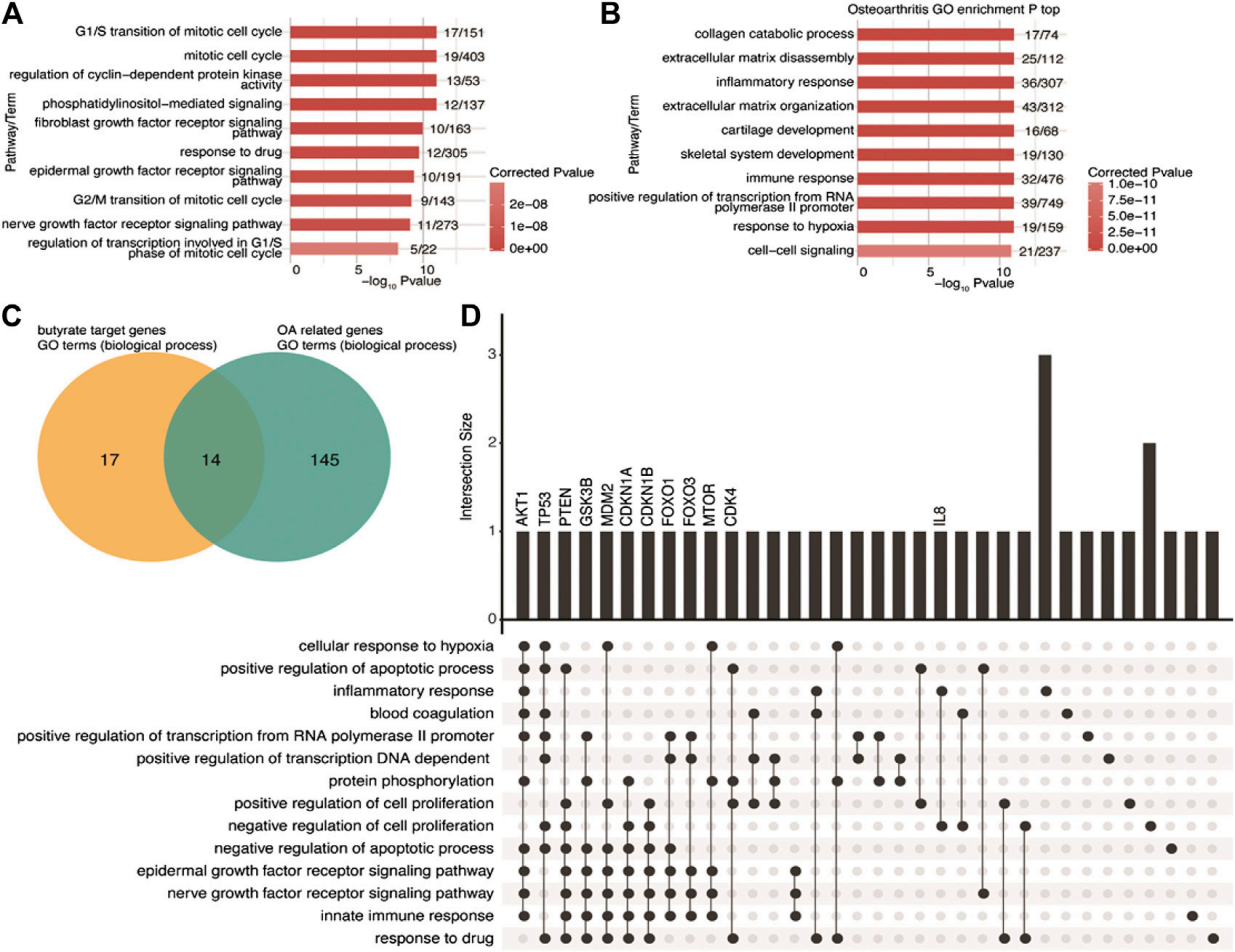
GO biological process terms shared between butyrate-targeted genes and OA-related genes **(A,B)** Top 10 most-enriched GO terms (biological process) for butyrate-targeted genes and OA-related genes. **(C)** Venn diagram showing the overlapping GO terms (biological process) enriched by both butyrate-targeted genes and OA-related genes. **(D)** Visualization of the intersection size of the butyrate-targeted genes involved in shared GO terms in **C**. GO, gene ontology; OA, osteoarthritis.

To investigate the functional features of butyrate-targeted gene sets and OA-related genes, we performed a GO enrichment analysis. A total of 31 GO biological process terms with corrected *p*-values less than 0.01 were identified (Supplementary Table S2) associated with butyrate-targeted gene sets, and the top 10 enriched terms are shown in Figure 1A. Using miRWalk 2.0 and MalaCards, 213 human OA-related genes were collected (Supplementary Table S3). GO enrichment analysis was then performed, and 159 GO biological process terms with corrected *p*-values less than 0.01 were identified (Figure 1B; Supplementary Table S4). We identified 14 GO terms that were shared between butyrate-targeted genes and OA (Figure 1C). The shared terms included the epidermal growth factor receptor signaling pathway, nerve growth factor receptor signaling pathway, negative regulation of cell proliferation, positive regulation of cell proliferation, negative regulation of the apoptotic process, positive regulation of the apoptotic process, innate immune response, and inflammatory response (Supplementary Table S1). The butyrate-targeted genes in these pathways were primarily associated with growth factors, cell survival, and the immune or inflammatory response. The UpSet intersection graph identified the butyrate-targeted hub genes that appeared in multiple pathways as *AKT1*, *TP53*, *PTEN*, *GSK3B*, *MDM2*, *CDKN1A*, *CDKN1B*, *FOXO1*, *FOXO3*, *MTOR*, and *CDK4* (Figure 1D). Among the overlapped terms, *IL8* and *TAC1* represent inflammatory response terms that are both butyrate-targeted genes and OA-related genes (Supplementary Table S6). Here, we focused on verifying the hypothesis that the protective effects of NaB on OA were mediated by PI3K/Akt/mTOR and related cross-talk signaling pathways *in vitro* and *in vivo*.

### Effects of NaB on Chondrocyte Viability

**FIGURE 2:**
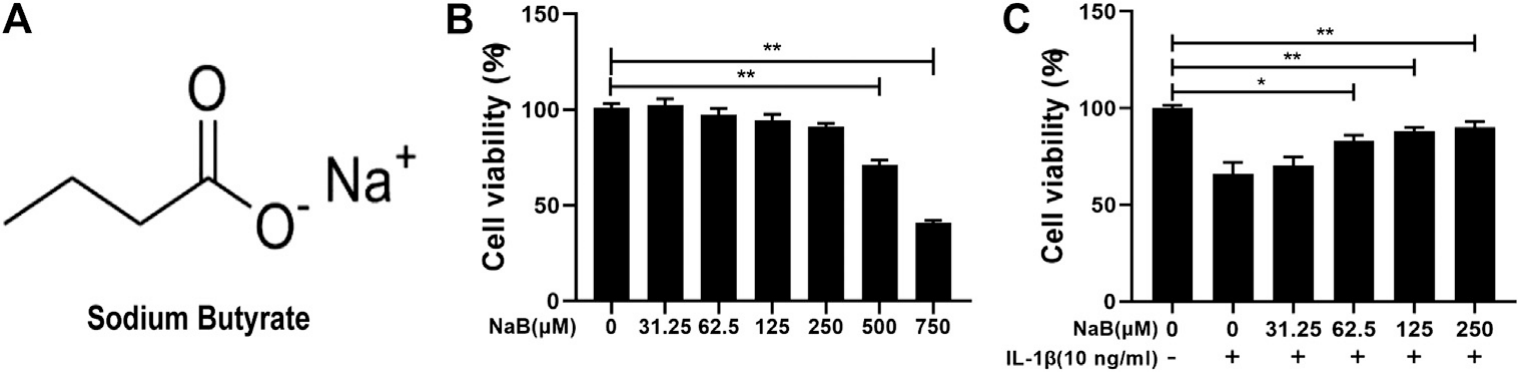
Effects of sodium butyrate (NaB) on the viability of chondrocytes. **(A)** The chemical structure of NaB. **(B)** Chondrocytes were examined with several NaB concentration gradients for 24 h, and the cytotoxic effects were assessed by the cell counting kit-8 (CCK-8) assay. **(C)** Chondrocytes were pretreated for 2 h with several concentration gradients of NaB and then stimulated with interleukin (IL)-1β for 24 h. The cytotoxic effects were detected by the CCK-8 assay. The data are presented as the mean ± S.D. Significant differences between the treatment and control groups were determined by one-way ANOVA; *p < 0.05, **p < 0.01; *n* = 3.

The chemical structure of NaB is shown in Figure 2A. To identify the optimal therapy concentration, we utilized cell counting kit-8 (CCK-8) assays to evaluate the effects of NaB on the viability of chondrocytes incubated with IL-1β. As demonstrated in Figures 2B,C, high concentrations (≥500 µM) of NaB significantly depressed cell viability, whereas low concentrations (≤250 µM) of NaB had no significant cytotoxic effects on chondrocytes after 24 h of treatment. Furthermore, compared with the control group, the dramatic inhibition of cell activity induced by IL-1β was reversed by NaB at lower concentrations (62.5, 125, and 250 µM) and in a dose-dependent manner. Therefore, 250 µM NaB was selected as the optimal concentration for further experiments, which was consistent with concentrations used in previously reported studies (Pirozzi et al., 2018).

### NaB Alleviates Articular Cartilage Degradation in a Mouse Model

To explore whether NaB possesses chondroprotective effects against OA *in vivo*, we generated a surgically induced ACLT mouse model to simulate the pathological process of OA. First, to determine the optimal treatment dose, we tested several NaB doses in a preliminary experiment, in which NaB was administered by oral gavage for 60 days after the surgery. Based on the HE staining, the HC thickness decreased in the vehicle and lower NaB dose groups (50 or 100 mg/kg) compared with those in the higher dose groups (150 or 200 mg/kg, Supplementary Figures S2A,C), which maintained a comparable HC thickness as observed in the sham group. Safranin O staining revealed that the lower NaB concentration groups showed pale Safranin O staining, which deepened in the higher concentration groups (Supplementary Figures S2B,D). In parallel with the International Cartilage Regeneration and Joint Preservation Society (ICRS) score for OA, these results indicated that the higher NaB concentration treatment levels could progressively promote proteoglycan synthesis and chondrogenesis. Compared with the 150 mg/kg NaB group, the highest dose group (200 mg/kg) demonstrated a reduced ability to promote proteoglycan generation in the articular cartilage (Supplementary Figures S2B,D). Therefore, the 150 mg/kg NaB dose was chosen as the optimal dose in the formal experiment.

**FIGURE 3:**
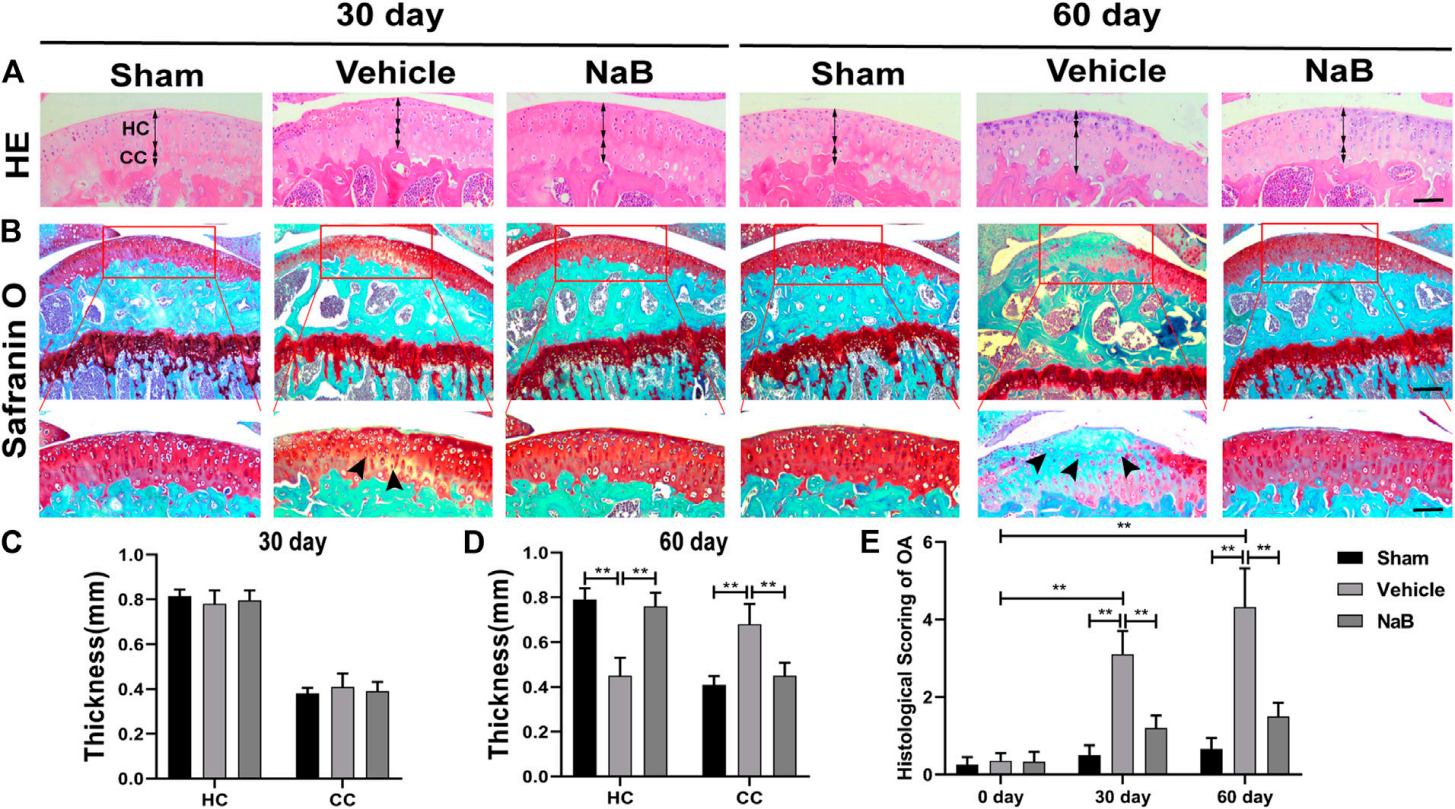
Sodium butyrate (NaB) prevents articular cartilage degeneration after ACLT in mice. **(A)** Hematoxylin and eosin (HE) staining; double-headed arrows represent the thickness of hyaline cartilage (HC) relative to calcified cartilage (CC). Scale bars, 100 µm. **(B)** Safranin O staining; solid arrows represent the loss of proteoglycans and cartilage degradation. Scale bar, 400 µm **(top panels)**; 200 µm **(bottom panels)**. **(C,D)** Variations in the thicknesses of HC and CC in distinct intervention groups and time points. **(E)** Histological osteoarthritis (OA) scoring for articular cartilage at distinct time points after surgery. *n* = 10 per group. The data are presented as the mean ± S.D. Significant differences between different groups were determined by one-way ANOVA **(C,D)** or Kruskal–Wallis H test **(E)**; *p < 0.05, **p < 0.01.

**FIGURE 4:**
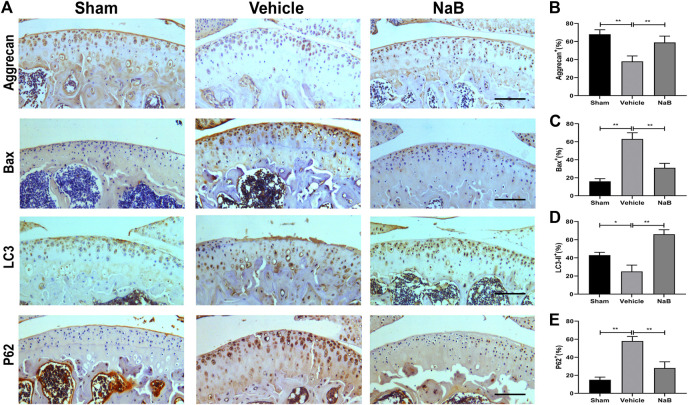
Sodium butyrate (NaB) normalizes the expression of aggrecan, Bax, LC3-II, and p62 in articular cartilage 60 days after the operation. **(A)** Immunostaining of aggrecan, Bax, LC3-II, and p62 after 60 days postoperation. **(B–E)** The quantification of cells with positive staining for aggrecan. **(B)**, Bax **(C)**, LC3-II **(D)**, and p62 **(E)**; scale bar, 100 μm, *n* = 10 per group. The data are presented as the mean ± S.D. Significant differences between the different groups were determined by one-way ANOVA; *p < 0.05, **p < 0.01.

Based on the histological analysis of Safranin O and HE staining performed in the formal experiment, we observed that the cartilage surface of the NaB group was smooth and remained red, similar to that in the sham group. Conversely, cartilage erosion, obvious hypocellularity, and significant proteoglycan loss could be observed in the vehicle group on postoperative day 60 (Figures 3B–D). NaB inhibited the rising tidemark compared with the vehicle group on postoperative day 60 (Figure 3A). Additionally, the ICRS score for OA decreased in the NaB group compared with the vehicle group, with no apparent difference between the NaB group and the sham group (Figure 3E). The immunohistochemical staining results showed a decrease in positive aggrecan staining in the cartilage of the vehicle group, which was markedly elevated in the NaB group, to levels similar to that observed in the sham group (Figures 4A,B). These results indicated that NaB has the ability to attenuate cartilage degradation *in vivo*.

### NaB Promotes Autophagy and Restrains Apoptosis *in Vivo*

To further explore the chondroprotective effects of NaB *in vivo*, the immunohistochemical staining of Bax, LC3-II, and P62 was performed at 60 days postoperation. The results showed that NaB normalized the expression levels of Bax and P62, which were pathologically elevated in the vehicle group (Figures 4A,C,E). In addition, NaB administration markedly increased LC3-II staining compared with both the sham and vehicle groups (Figures 4A,D). In conclusion, the activation of autophagy and the decrease in apoptosis observed in articular cartilage likely mediate the protective effects of NaB against OA.

### NaB Restores the Interleukin-1β-Induced Blockade of Autophagy and Autophagic Flux in Chondrocytes

**FIGURE 5:**
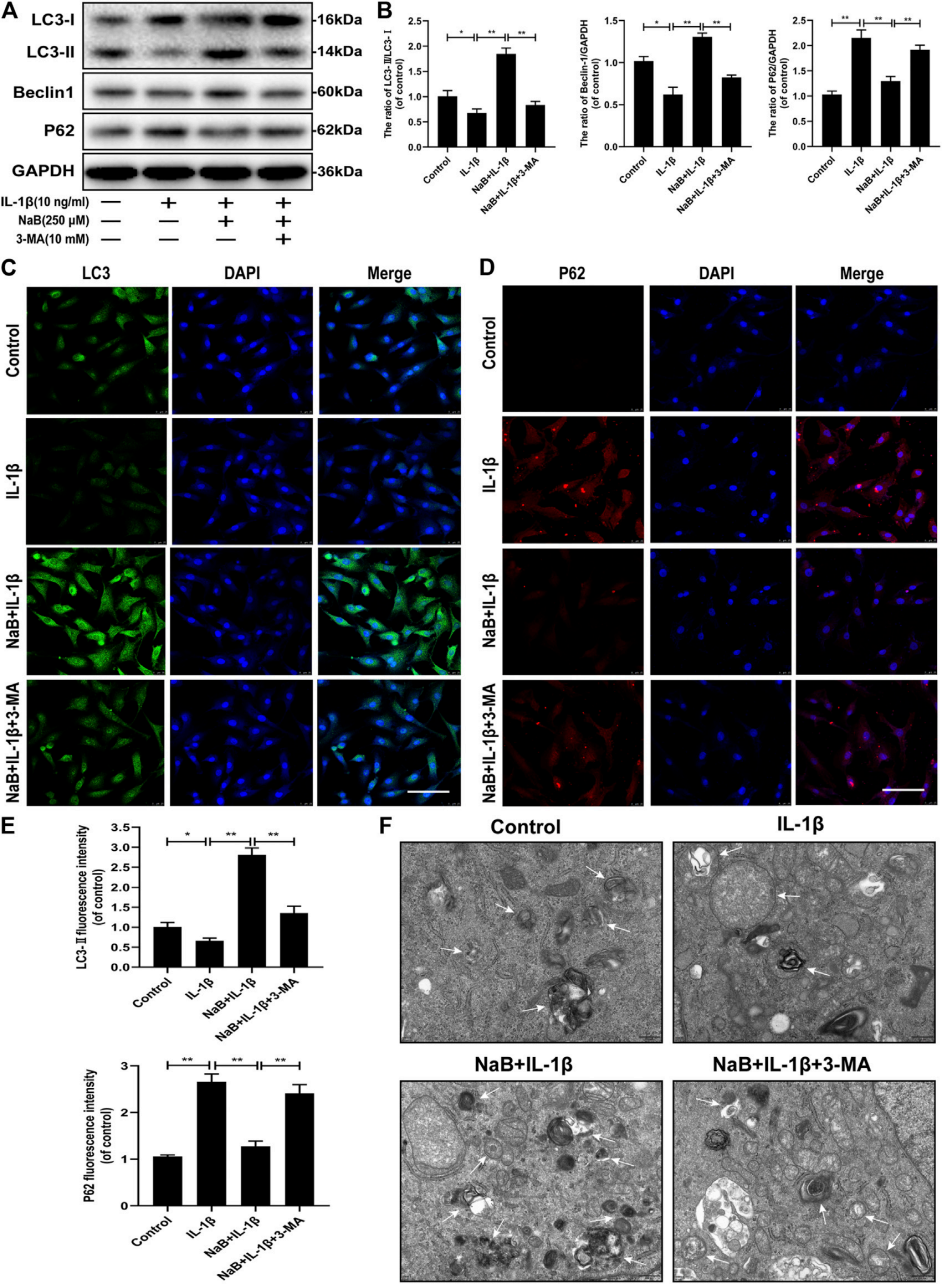
Sodium butyrate (NaB) restores the interleukin (IL)-1β-induced blockade of autophagy and autophagic flux in chondrocytes. Untreated (DMEM-F12, 10% FBS) chondrocytes were compared with chondrocytes treated with IL-1β alone, NaB combined with IL-1β, or IL-1β, NaB, and 3-methyladenine (3-MA, 10 mM) for 24 h. Protein samples from each intervention group were collected. **(A,B)** The protein expression levels of LC3, Beclin-1, and p62 were detected by western blotting. The immunofluorescent detection of LC3-II **(C)** and p62 **(D)** in chondrocytes **(E)** Quantitative analysis of LC3-II and p62 fluorescent intensity (the green signal represents LC3-II, the red signal represents p62, scale bar: 100 µm). **(F)** Transmission electron microscopy (TEM) images showing autophagic changes in chondrocytes; white arrow: autophagolysosome and autophagosome; scale bar, 500 nm. The data are presented as the mean ± S.D. Significant differences between the different groups were determined by one-way ANOVA; *p < 0.05, **p < 0.01; *n* = 3.

Autophagy plays a critical role in the mediation of energy metabolization and cellular homeostasis in chondrocytes. To explore whether NaB treatment could promote the activation of autophagy and enhance autophagic flux in chondrocytes, autophagic indicators, including the LC3-II/LC3-I ratio, Beclin-1, and SQSTM1/p62, were detected by western blotting. As shown in Figures 5A,B, the ratio of LC3-II/LC3-I and the expression level of Beclin-1 was predominantly suppressed after 24 h of treatment with IL-1β alone. All of these negative effects were reversed after pretreatment with NaB. As expected, the accumulation of IL-1β-induced SQSTM1/p62 expression was reduced by NaB intervention. To confirm these findings, a fluorescently labeled anti-LC3-II antibody (a marker of the autophagosome) showed an increase in fluorescence intensity following the NaB treatment of chondrocytes compared with chondrocyte treated with IL-1β alone. However, the opposite phenomenon was observed for the immunofluorescent analysis of SQSTM1/p62 expression (Figures 5C–E).

To further explore the NaB-induced effects on autophagy, we used TEM to observe the presence of autophagosomes and autophagolysosomes in various intervention groups, which represents a standard method for observing autophagy activation. Consistent with the previous results, increased autophagosomes and autophagolysosomes were observed in the cytoplasm of NaB-treated chondrocytes compared with those in the control and IL-1β treatment groups (Figure 5F). In conclusion, these data validated that autophagy and autophagic flux could be effectively enhanced by NaB treatment, which can be partially inhibited by treatment with 3-methyladenine (3-MA).

### NaB Alleviates Interleukin-1β-Induced ECM Degradation and Inflammatory Cytokine Expression *via* Autophagy in Chondrocytes

**FIGURE 6:**
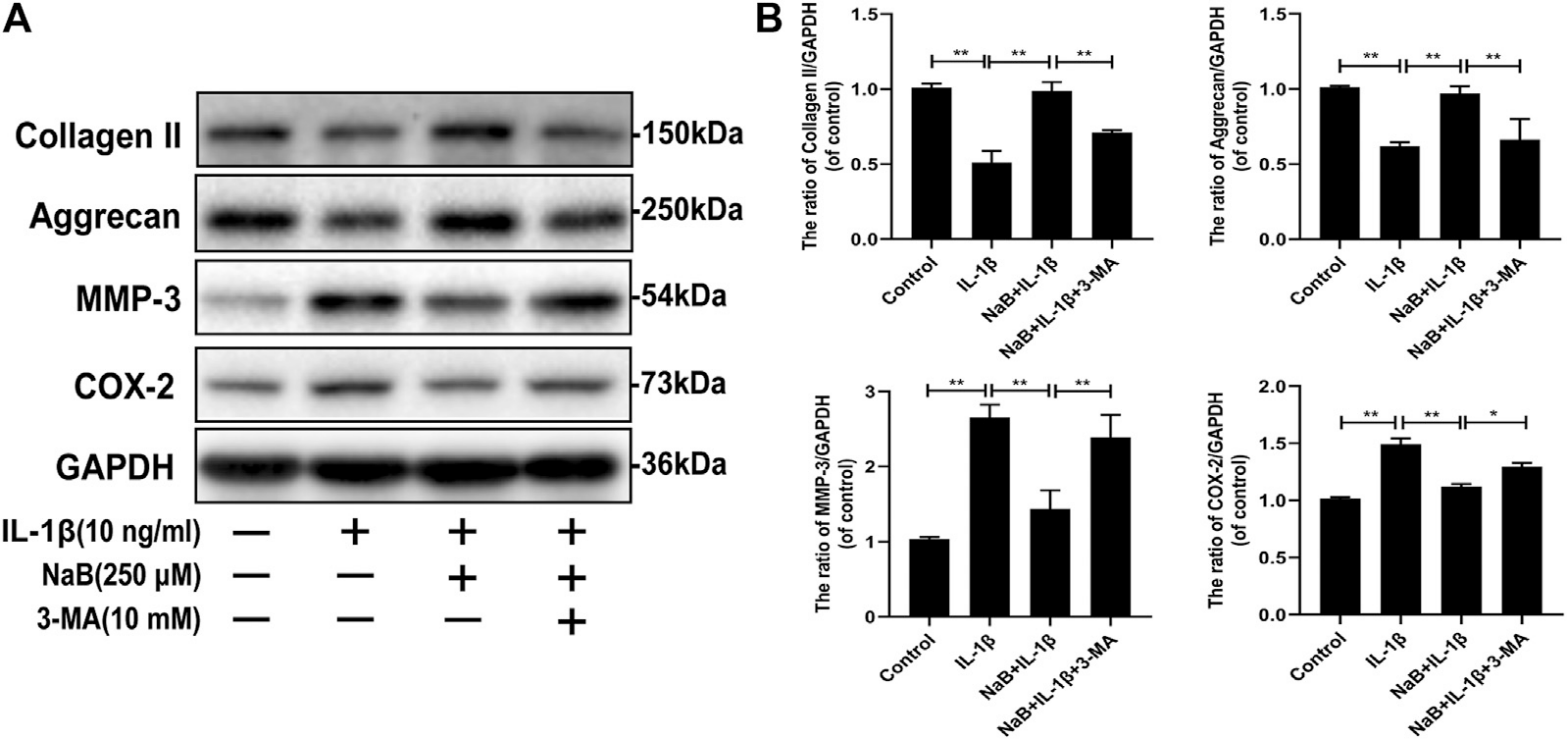
Sodium butyrate (NaB) alleviated interleukin (IL)-1β-induced extracellular matrix (ECM) degradation and inflammation in chondrocytes. Chondrocyte treatments were the same as above **(A,B)** Chondrocyte expression of collagen II, aggrecan, matrix metalloproteinase (MMP)-3, and cyclooxygenase (COX)-2 proteins detected by western blotting. The data are presented as the mean ± S.D. Significant differences between groups were determined by one-way ANOVA; *p < 0.05, **p < 0.01; *n* = 3.

Collagen II and aggrecan, which are cartilage-specific markers, and matrix metalloproteinase-3 (MMP-3) and cyclooxygenase 2 (COX-2), which are inflammatory markers, were measured *via* western blotting. As shown in Figures 6A,B, IL-1β treatment prominently reduced collagen II and aggrecan synthesis but increased MMP-3 and COX-2 expression. However, the harmful effects induced by IL-1β were reversed by NaB pretreatment. Interestingly, we observed that the chondrocyte-protective effects of NaB were partially abrogated by the autophagy inhibitor 3-MA (Figures 6A,B), which indicated that the chondroprotective effects of NaB were partly mediated by autophagy. These results indicated that NaB exerted a chondroprotective effect through the promotion of ECM synthesis and anti-inflammatory effects.

### NaB Suppresses Interleukin-1β-Induced Apoptosis *via* Autophagy in Chondrocytes

**FIGURE 7:**
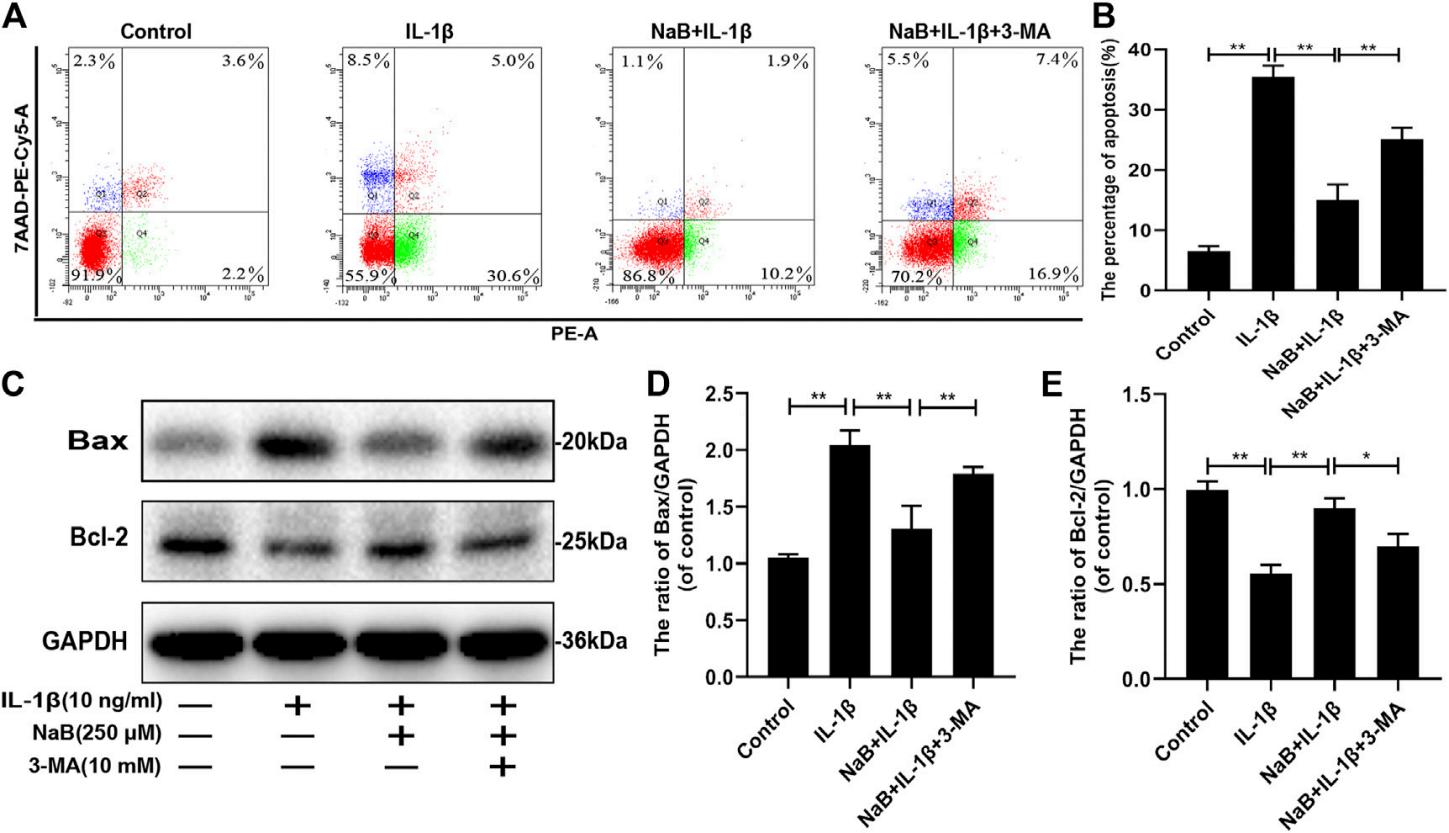
Sodium butyrate (NaB) suppresses interleukin (IL)-1β-induced apoptosis in chondrocytes. The chondrocytes treatments were the same as described above. **(A,B)** Apoptotic rates of different groups were determined using a flow apoptosis kit. **(C–E)** The protein expression of Bax and Bcl-2 was detected by western blotting. The data are presented as the mean ± S.D. Significant differences between groups were determined by one-way ANOVA; *p < 0.05, **p < 0.01; *n* = 3.

To explore the effect of NaB on chondrocyte apoptosis induced by IL-1β, western blot and flow cytometric analyses were performed. As shown in Figures 7A,B, the percentage of apoptotic chondrocytes increased upon IL-1β treatment. Similarly, the expression of the pro-apoptotic protein Bax was remarkably upregulated by IL-1β treatment compared with that in other groups (Figures 7C–E). In contrast, these changes were attenuated by NaB intervention, as demonstrated by reduced chondrocyte apoptosis, which was consistent with the downregulated expression of Bax and the upregulated expression of the anti-apoptotic protein Bcl-2. In addition, we found that the anti-apoptotic effects of NaB were partly abolished by 3-MA treatment (Figures 7A–E). Taken together, the above results indicated that NaB exerts anti-apoptotic effects, which are likely mediated by autophagy activation.

### NaB Prevents Interleukin-1β-Induced Reactive Oxygen Species and Cell Cycle Arrest *via* Autophagy in Chondrocytes

**FIGURE 8:**
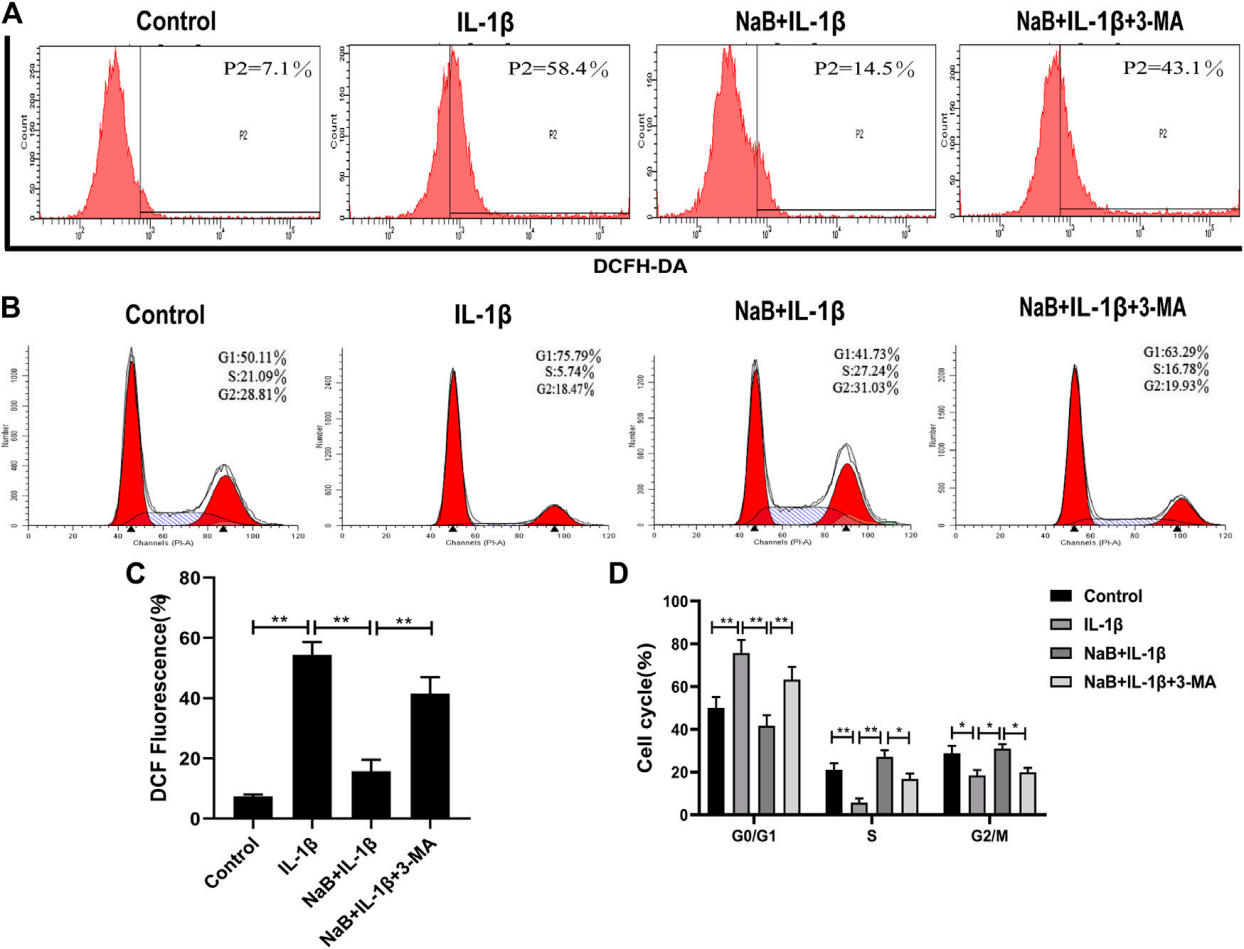
Sodium butyrate (NaB) prevents IL-1β-induced reactive oxygen species (ROS) and cell cycle arrest in chondrocytes. The chondrocytes treatments were the same as above. Cellular ROS was assessed by flow cytometry **(A)** and quantified **(C)**. Changes in the chondrocyte cell cycle were analyzed by flow cytometry among four treatment groups **(B)** and quantified **(D)**. The data are presented as the mean ± S.D. Significant differences between groups were determined by one-way ANOVA; *p < 0.05, **p < 0.01; *n* = 3.

To investigate whether NaB had any effects on the production of ROS and cell cycle arrest in chondrocytes, we used flow cytometry to detect intracellular ROS production and DNA concentrations. We then explored the effects of the autophagy inhibitor 3-MA on these processes. As shown in Figures 8A,C, the intracellular generation of ROS was dramatically enhanced by IL-1β treatment. However, treatment with NaB was able to effectively reduce ROS generation.

In addition, the chondrocytes in the IL-1β-induced group were arrested at the G1 phase, with fewer chondrocytes in the S phase compared with the control group (Figures 8B,D). Upon the addition of NaB, the proportion of haploid cells was reduced, with more chondrocytes in the S phase and fewer chondrocytes in the G1 phase (Figures 8B,D). Confirming these results, pretreatment with 3-MA resulted in an obvious increase in ROS generation and cell cycle arrest compared with the NaB treatment group (Figures 8A–D). These data suggested that NaB effectively inhibited the IL-1β-induced increase in ROS and cell cycle arrest in chondrocytes, and the effects of NaB could be partially arrested by 3-MA treatment.

### NaB Enhances Autophagic Flux by Modulating the PI3K/Akt/mTOR Signaling Pathway

**FIGURE 9:**
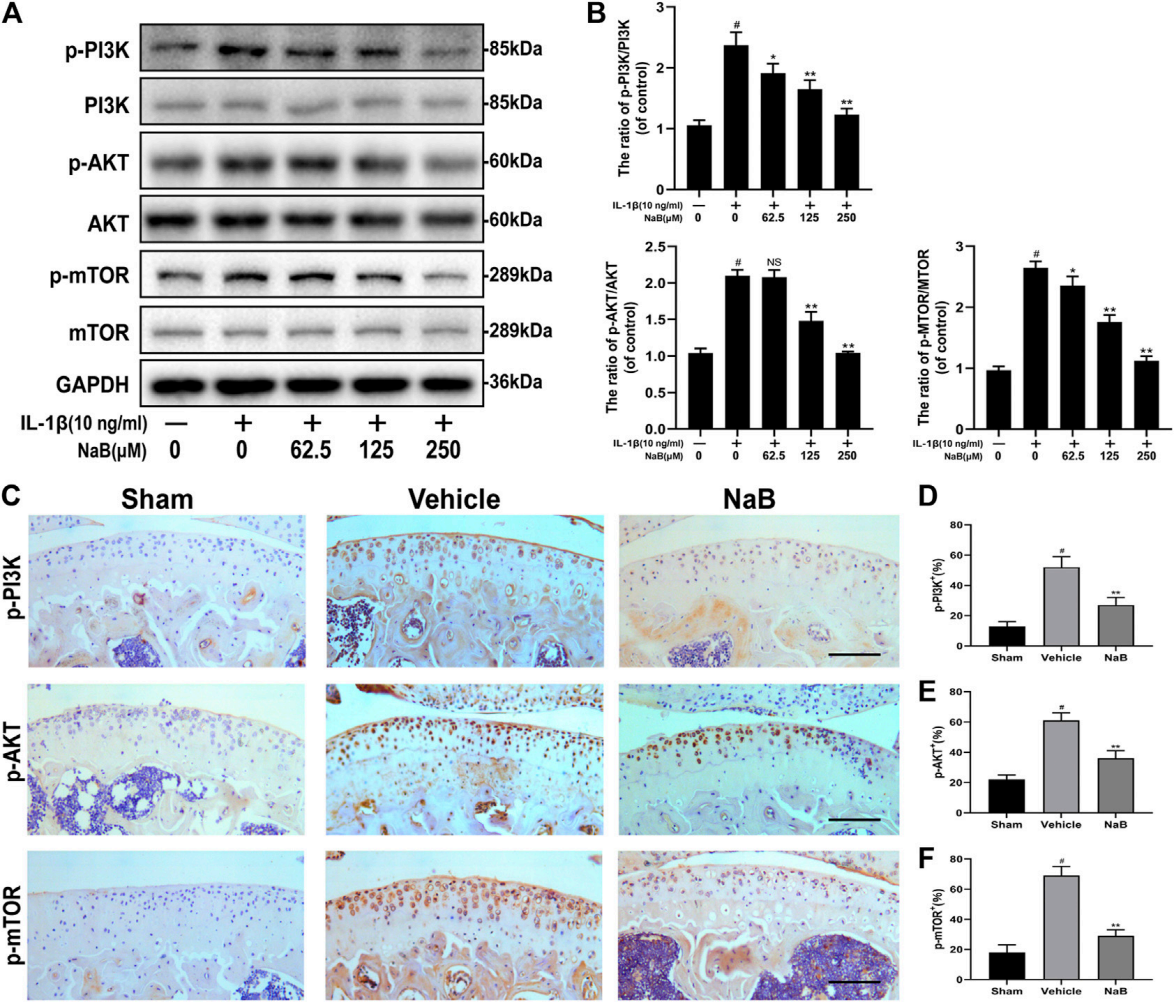
Sodium butyrate (NaB) arrests the PI3K/Akt/mTOR signaling pathway *in vitro* and *in vivo*. Chondrocytes were pretreated for 2 h with several concentration gradients of NaB and were then stimulated with IL-1β for 24 h **(A,B)** The protein expression levels of PI3K, p-PI3K, Akt, *p*-Akt, mTOR, and *p*-mTOR in chondrocytes were assessed (*n* = 3). **(C)** Immunostaining of p-PI3K, *p*-Akt, and *p*-mTOR 60 days postoperation and quantification **(D–F)**; scale bar, 100 μm; *n* = 10 per group. The data are presented as the mean ± S.D. Significant differences between groups were determined by one-way ANOVA; *p < 0.05 and **p < 0.01 vs. IL-1β-induced group or the vehicle group; ^#^ *p* < 0.01 vs. control group or the sham group; NS (not significant) vs. control group or the sham group. PI3K, phosphoinositide 3-kinase; Akt, protein kinase B; mTOR, mammalian target of rapamycin; IL-1β, interleukin-1β.

To further explore the molecular mechanism of NaB-induced autophagy in IL-1β-induced chondrocytes, key components of the PI3K/Akt/mTOR pathway were analyzed by western blot and immunohistochemical staining. As shown in Figures 9A,B, the expression levels of phosphorylated PI3K, Akt, and mTOR were dramatically upregulated by IL-1β administration compared with those in the control group. By contrast, NaB markedly diminished the IL-1β-induced phosphorylation of PI3K, Akt, and mTOR in a concentration-dependent manner. Consistent with the above data, the immunohistochemical staining results showed the elevation of positive staining for p-PI3K, p-Akt, and p-mTOR in the cartilage of the vehicle group, which was markedly decreased in the NaB group, to levels similar to those in the sham group (Figures 9C–F). In conclusion, these findings suggested that the suppression of PI3K/Akt signaling, which are key upstream inhibitors of mTOR, is a potential mechanism through which NaB improves autophagy levels and autophagic flux in OA.

## Discussion

In recent years, emerging evidence has indicated that GMB dysbiosis and changes in metabolite production are closely related to OA pathogenesis (Kalinkovich and Livshits, 2019; Liu et al., 2019). Different from traditional drug therapy, strategies that target homeostasis maintenance among GMB or the regulation of GMB metabolites are expected to become attractive therapy options for OA (Berthelot et al., 2019; Boer et al., 2019). NaB, a component of GMB metabolites, has been used to treat various diseases, ranging from enteritis to tumors and bone metabolism diseases. To the best of our knowledge, the present study is the first to demonstrate that NaB treatment can alleviate the IL-1β-induced inflammation, ROS, cell cycle arrest, and apoptosis in chondrocytes, an effect that appears to be mediated by the activation of autophagy flux in chondrocytes *via* the regulation of the PI3K/Akt/mTOR signaling pathway. In addition, the administration of NaB by oral gavage exerted potential therapeutic effects on articular cartilage in an ACLT mouse model.

Healthy chondrocytes that are capable of responding to microenvironmental changes in the surrounding cartilage matrix play important roles in the maintenance of the dynamic metabolism balance of the ECM. During the pathological process of OA, however, this balance is disrupted by elevated inflammatory mediators and MMPs. Previous studies have confirmed that MMPs and a disintegrin and metalloproteinase with thrombospondin motifs (ADAMTs) can degrade collagen II and proteoglycans in the ECM, and the suppression of these enzymes can protect the articular cartilage from degeneration (Verma and Dalal, 2011). In the present study, NaB treatment not only restored the expression of chondrocyte anabolic markers, such as collagen II and aggrecan, but also suppressed the pathological elevation of the catabolic marker MMP-3, which was stimulated by IL-1β treatment. However, we also found that pro-inflammatory cytokines, such as COX-2, which was responsible for the destruction of articular cartilage (Malemud et al., 2003), were also reduced by NaB treatment in IL-1β-treated chondrocytes. The chondroprotective effects of NaB could be partially abrogated by 3-MA, which indicated that the protective effects of NaB were partly mediated by autophagy. In addition, the administration of NaB by oral gavage to the surgically induced ACLT mouse model suppressed the advancement of CC and enhanced the deposition of proteoglycans, as observed on postoperative day 60, and was combined with the normalization of ICRS scores in the NaB group. In conclusion, these observations demonstrated that NaB exhibits protective effects in chondrocytes that are partially mediated by the reinforcement of autophagy.

Articular cartilage is an avascular tissue with a low rate of chondrocyte proliferation; thus, chondrocytes are forced to adapt to a low-oxygen environment by becoming quiescent (Zhang F.-J. et al., 2015). Previous studies have confirmed that age-related disorders in ROS production, combined with the impaired antioxidant capacity of chondrocytes, can lead to the accumulation of impaired DNA (Grishko et al., 2009), playing a vital role in chondrocyte apoptosis (Zheng et al., 2018a). The elevated rate of apoptosis in chondrocytes not only promotes the production of matrix-degrading enzymes but also, and perhaps more importantly, accelerates cartilage degradation in OA (Hwang and Kim, 2015). As expected, the presence of IL-1β dramatically increased ROS levels and the accumulation of impaired DNA, in addition to increasing the apoptosis rate of chondrocytes, which inhibited ECM and reduced chondrocyte proliferation. In agreement with previous studies, the expression of pro-apoptotic factors, such as Bax, was upregulated, whereas the anti-apoptotic Bcl-2 was downregulated in IL-1β-treated chondrocyte. However, NaB treatment not only reversed pathologically elevated ROS levels and reversed cell cycle arrest and apoptosis rates in chondrocytes but also normalized the expression of Bax in the ACLT mouse model, as indicated by immunohistochemical staining. We observed that pretreatment with 3-MA weakened the chondroprotective effects of NaB. Interestingly, several studies have revealed that NaB induces autophagic apoptosis in tumor cell lines (Zhang et al., 2016; Huang et al., 2019). The differential effects of NaB treatment may be due to differences in doses, treatment times, or cell types. Taken together, these data confirmed that NaB exhibited an anti-apoptotic effect in IL-1β-treated chondrocytes by attenuating oxidative stress and DNA lesions, which were partly mediated by autophagy.

Autophagy is a cellular homeostasis mechanism that plays an important role in the proliferation, differentiation, and maturation of chondrocytes (Shapiro et al., 2014) and is closely related to the pathological process of OA (Barranco, 2015). Furthermore, the expression of OA-associated genes, such as *ACAN*, *MMP*-*13*, and *ADAMTS*-*5*, can be reversed by autophagy through the regulation of ROS and apoptosis (Sasaki et al., 2012). Therefore, we explored the chondroprotective effects of NaB treatment on chondrocyte autophagy. Our results showed that the suppressed expression of LC3-II/LC3-I and Beclin-1 and the upregulation of p62 protein could be reversed by NaB treatment compared with the expression levels in IL-1β-induced chondrocytes. In addition, the TEM imaging results showed that NaB treatment enhanced the generation of autophagic flux and autolysosomes, which were arrested by IL-1β treatment in chondrocytes. Furthermore, we established an ACLT mouse model to evaluate the effects of NaB on autophagy during cartilage degeneration, which was assessed by immunohistochemical staining. In line with the above results, LC3-II staining was significantly increased by NaB administration compared with the levels observed in the sham and vehicle groups on postoperative day 60. In contrast, a lower level of p62 protein was observed in NaB-treated chondrocytes and in the ACLT mouse model, implying that the suppression of autophagic flux was restored by NaB treatment. The NaB-induced autophagy and autophagic flux could be markedly abrogated by pretreatment with 3-MA in chondrocytes. Taken together, these results validated that the autophagy and autophagic flux could be effectively restored by NaB treatment and partially inhibited by treatment with 3-MA.

The key regulator of autophagy is mTOR, which is regulated by several upstream signaling pathways, such as the 5′ AMP-activated protein kinase (AMPK) and the PI3K/Akt pathways (Yang and Guan, 2007). Combined with the results of our network pharmacology analysis of NaB, we speculated that PI3K/AKT inhibition might be responsible for the NaB-induced autophagy effects observed in chondrocytes. AMPK is typically stimulated under circumstances of energy and nutrient stress, and the inhibition of PI3K/Akt primarily occurs in response to chemical, physical, or genetic factors (Bujak et al., 2015; Ha et al., 2015). Furthermore, the PI3K/Akt pathway is also responsible for the regulation of apoptosis and inflammation-related factors in chondrocytes (Xue et al., 2017). Our results indicated that NaB treatment downregulated the expression of phosphorylated PI3K, Akt, and mTOR compared with the levels in the IL-1β treatment group in a dose-dependent manner. Similar results were obtained from the immunohistochemical staining of phosphorylated PI3K, Akt, and mTOR *in vivo*. Certainly, if we performed RNA interference or gene knockdown instead of molecular inhibition, the results would likely be even more persuasive. Although this is a limitation of our study, the chondroprotective effects of NaB on autophagy were validated, and a clear regulatory mechanism exists between NaB and autophagy in OA that requires further exploration. Taken together, all of these results suggested that the inhibition of PI3K/Akt signaling, a key upstream inhibitor of mTOR, may represent the mechanism through which NaB restores autophagic flux in chondrocytes.

In summary, we demonstrated that NaB attenuated OA progression, including the amelioration of IL-1β-induced inflammation, ROS production, cell cycle arrest, and apoptosis in chondrocytes, and reduced articular cartilage degeneration in a mouse ACLT model by restoring impaired autophagy and autophagic flux, mediated by the PI3K/Akt/mTOR pathway *in vitro* and *in vivo.* These findings provide insight into potential strategies that target NaB for the prevention and treatment of OA.

## Data Availability

The original contributions presented in the study are included in the article/Supplementary Material, further inquiries can be directed to the corresponding authors.
