# Supplementary figures and images for: Microbial Metabolite Sodium Butyrate Attenuates Cartilage Degradation by Restoring Impaired Autophagy and Autophagic Flux in Osteoarthritis Development
Source: Front Pharmacol. 2021 Apr 9;12:659597. doi: 10.3389/fphar.2021.659597 (PMC8062861; doi:10.3389/fphar.2021.659597)

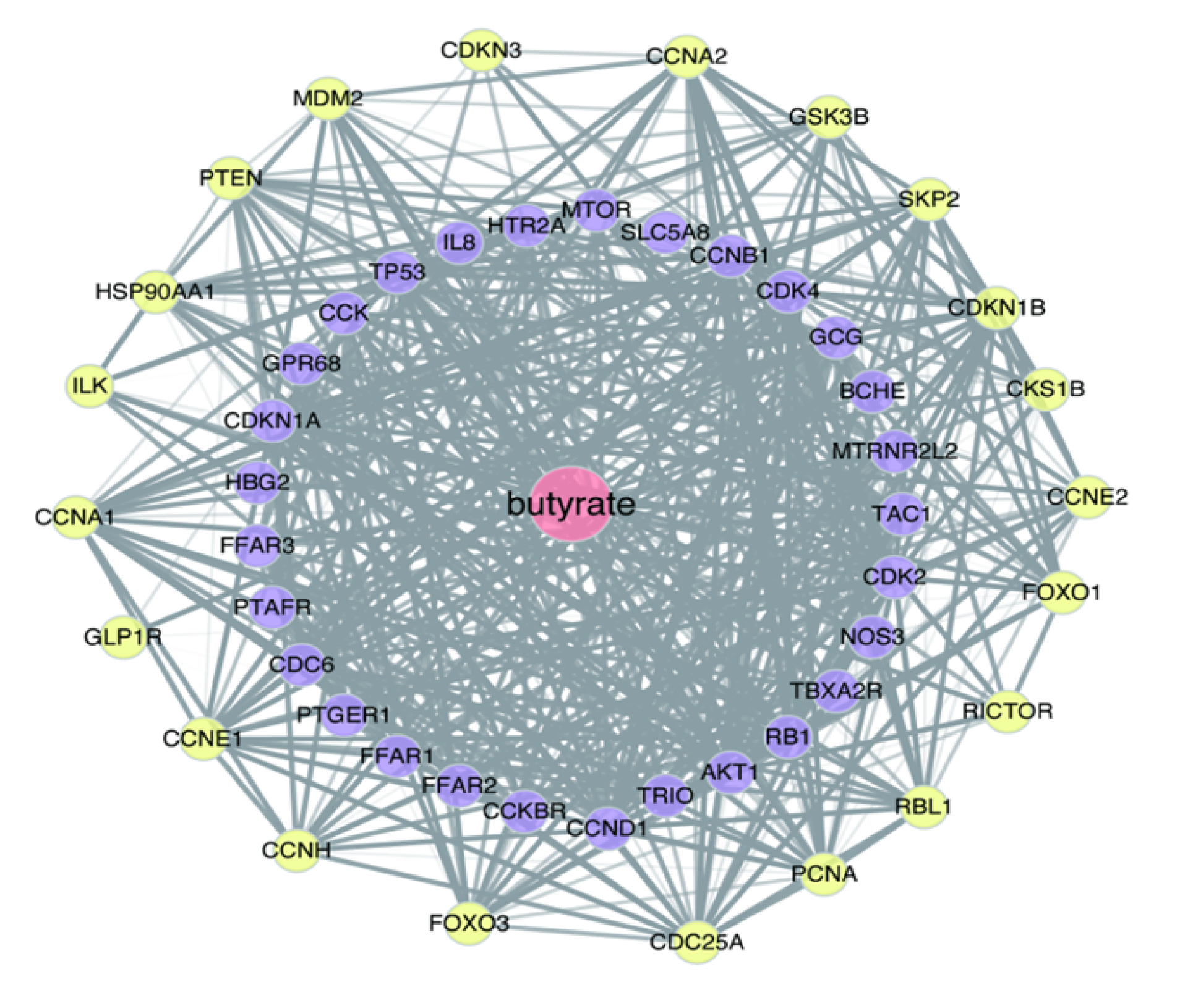

Supplement: Supplementary file 1 [file image1.tif]

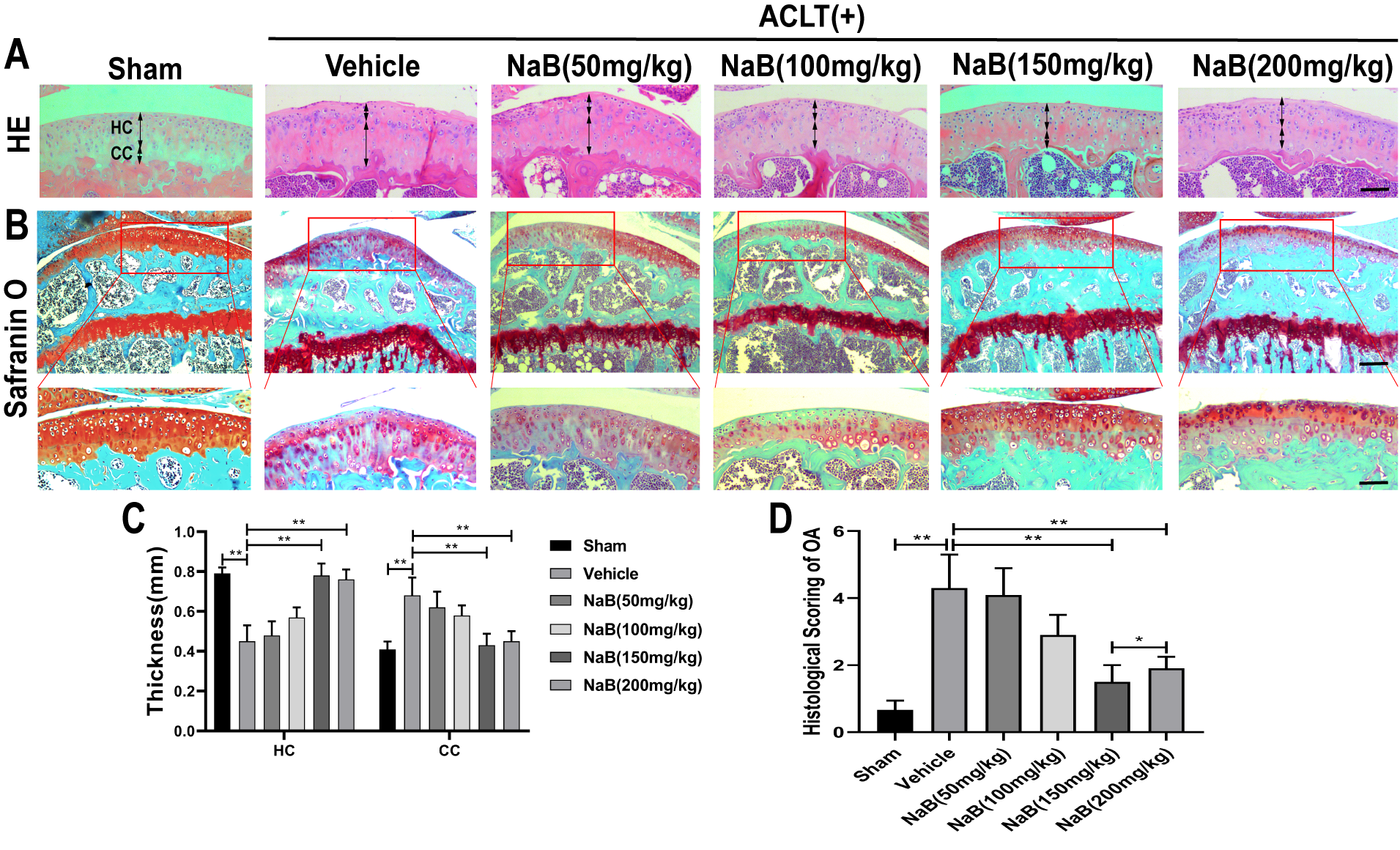

Supplement: Supplementary file 2 [file image2.tif]
