# Supplementary material for: Microbial Metabolite Sodium Butyrate Attenuates Cartilage Degradation by Restoring Impaired Autophagy and Autophagic Flux in Osteoarthritis Development
Source: Front Pharmacol. 2021 Apr 9;12:659597. doi: 10.3389/fphar.2021.659597 (PMC8062861; doi:10.3389/fphar.2021.659597)
Supplement: Supplementary file 3 [file table1.docx]

**Supplementary table-1**: GO biological process terms of butyrate-targeted genes shared by OA

| **Term** | **ID** | **Corrected P-Value** | **Input** |
| --- | --- | --- | --- |
| response to drug | GO:0042493 | 1.24E-09 | TBXA2R, CCNB1, TP53, CCND1, CDKN1B, CDKN1A, GSK3B, PTEN, HTR2A, BCHE, MDM2, CDK4 |
| epidermal growth factor receptor signaling pathway | GO:0007173 | 2.63E-09 | AKT1, CDKN1B, CDKN1A, GSK3B, FOXO3, PTEN, FOXO1, MTOR, MDM2, RICTOR |
| nerve growth factor receptor signaling pathway | GO:0048011 | 4.07E-09 | TRIO, PTEN, CDKN1B, CDKN1A, GSK3B, FOXO3, AKT1, FOXO1, MTOR, MDM2, RICTOR |
| protein phosphorylation | GO:0006468 | 3.30E-06 | CCNB1, CDKN1A, CCNE1, ILK, GSK3B, AKT1, CDK4, MTOR, CCNH |
| negative regulation of cell proliferation | GO:0008285 | 6.42E-06 | NOS3, TP53, CDKN3, CDKN1B, CDKN1A, PTEN, BCHE, IL8, CDC6 |
| positive regulation of cell proliferation | GO:0008284 | 1.62E-05 | CCKBR, CDKN1B, ILK, PTEN, CDK2, CDK4, CCK, HTR2A, MDM2 |
| cellular response to hypoxia | GO:0071456 | 1.78E-05 | MTOR, AKT1, MDM2, TP53, CCNB1 |
| negative regulation of apoptotic process | GO:0043066 | 3.33E-05 | TP53, GCG, PTEN, CDKN1B, CDKN1A, GSK3B, AKT1, FOXO1, MDM2 |
| innate immune response | GO:0045087 | 3.94E-05 | PTEN, CDKN1B, CDKN1A, GSK3B, FOXO3, AKT1, FOXO1, MTOR, HSP90AA1, MDM2, RICTOR |
| positive regulation of transcription, DNA-dependent | GO:0045893 | 0.000314402 | CCNA2, CCNE1, FOXO1, RB1, ILK, FOXO3, CDK2, TP53 |
| positive regulation of apoptotic process | GO:0043065 | 0.000529488 | TRIO, TP53, PTEN, AKT1, CDK4, CCK |
| inflammatory response | GO:0006954 | 0.000642419 | TBXA2R, TAC1, AKT1, PTAFR, GPR68, IL8 |
| positive regulation of transcription from RNA polymerase II promoter | GO:0045944 | 0.003507724 | TP53, RBL1, RB1, GSK3B, FOXO3, AKT1, FOXO1, CCNH |
| blood coagulation | GO:0007596 | 0.005030437 | TBXA2R, NOS3, TP53, HBG2, AKT1, CDK2 |
